# Electrochemical Oxidation of l-selenomethionine and Se-methylseleno-l-cysteine at a Thiol-Compound-Modified Gold Electrode: Its Application in a Flow-Through Voltammetric Sensor

**DOI:** 10.3390/s17020383

**Published:** 2017-02-16

**Authors:** Lai-Hao Wang, Yu-Han Zhang

**Affiliations:** Department of Medical Chemistry, Chia Nan University of Pharmacy and Science, 60 Erh-Jen Road, Section 1, Rende, Tainan 71743, Taiwan; je20120108@gmail.com

**Keywords:** thiol-modified gold electrodes, amperometric detection, l-selenomethionine, Se-methylseleno-l-cysteine

## Abstract

A flow-electrolytic cell that consists of a bare gold wire or of different thiol-compound-modified gold electrodes (such as 2,4-thiazolidinedione, 2-mercapto-5-thiazoline, 2-mercaptothiazoline, l-cysteine, thioglycolic acid) was designed to be used in a voltammetric detector to identify l-selenomethionine and Se-methylseleno-l-cysteine using high-performance liquid chromatography. Both l-selenomethionine and Se-methylseleno-l-cysteine are more efficiently electrochemically oxidized on a thiol/gold than on a bare gold electrode. For the DC mode, and for measurements with suitable experimental parameters, a linear concentration from 10 to 1600 ng·mL^−1^ was found. The limits of quantification for l-selenomethionine and Se-methylseleno-l-cysteine were below 10 ng·mL^−1^. The method can be applied to the quantitative determination of l-selenomethionine and Se-methylseleno-l-cysteine in commercial selenium-containing supplement products. Findings using high-performance liquid chromatography with a flow-through voltammetric detector and ultraviolet detector are comparable.

## 1. Introduction

Rezvanfar et al. [1] reported that the immunomodulator drug (IMOD) they tested was an electromagnetically-treated, selenium (Se)-based, herbal medicine with antioxidant properties and that it prevented the complications of polycystic ovary. The effects of bacteriostasis in vitro of Se-enriched herbal medicine on *Escherichia coli* and *Salmonella* have been studied [2,3]. Therefore, it is used to prepare a high-energy composite peptide selenoprotein nutrient solution [4]. Selenium is an essential component of several antioxidant enzymes in animal taxonomic groups, and exposure to too much or too little Se can negatively affect an organism [5]. It has been reported that the concentrations for toxicity, bioavailability and reactivity of Se depend on its chemical forms and concentration. Medicinal plants enriched with anticarcinogenic Se compounds can be used to improve public health. Se in plants exists predominantly in organic forms, such as selenoamino acids (selenocysteine, selenocysteine, selenomethionine and Se-methylselenocysteine) and in trace quantities in inorganic Se species. Hence, accurate and precise analytical methodologies for determining Se are required to obtain the correct chemical forms and concentrations [6,7,8].

Several methods for determining Se have been developed over the years: fluorescence spectrophotometry [9,10], microscopic identification [11], atomic absorption spectrometry [12,13], inductively-coupled plasma atomic emission spectroscopy (ICP-AES) [14,15], chromatographic separation coupled to high-performance liquid chromatography inductively-coupled plasma mass spectrometry (HPLC-ICP-MS) [16,17,18,19,20] and electrochemical techniques [21,22,23,24,25,26,27,28,29,30,31]. Generally, the analytical techniques used to determine are HPLC-ICP-MS. The above-mentioned analytical methods have low limits of detection, selectivity and sensitivity, but they were widely used in different matrices. The voltammetric technique that we propose here, however, is inexpensive, excellently sensitive and selective and used primarily for speciation analysis and mechanism research. The electrochemical behavior of selenocysteine (SeCyst) has previously been studied on Au-, silver (Ag)- and silver nitrate (AgNO_3_)-modified carbon paste electrodes [21]. Electrochemical oxidation of selenoamino acids (selenocysteine and selenomethionine) on gold electrodes [22] and of selenocysteine on Se-Au film-modified glassy carbon electrodes [23] have also been investigated. Since the mainstream development of gold-alkanethiol self-assembled monolayers (SAMs) in the early 1980s, they have found widespread utility in the fabrication of chemical and biological sensors [24,25,26,27,28,29,30,31]. The least expensive and the simplest way to synthesize thin films of a variety of compounds is based on using various SAM substrates on gold surfaces. However, our review of the literature revealed no research on the electrochemical oxidation of l-selenomethionine or Se-methylseleno-l-cysteine on the five kinds of thin-film modified groups of gold electrodes. The modified groups included thioheterocycles groups (2, 4-thiazolidinedione, 2-mercapto-5-thiazoline and 2-mercaptothiazoline) and thiol groups (l-cysteine and thioglycolic acid). It has been reported [32] that many sulfuric compounds, e.g., sulfide, disulfide and mercaptan, can self-assemble as thin and sequential monolayers on a metal substrate. Thioctic acid can modify a gold surface through its S-S bond. It follows that SAMs can be exploited for their directed conjugation and incorporation of biocomponents and, therefore, surface functionalization [33]. The charge transfer across the Se-Au bond is less pronounced than that in the S-Au bond, which indicates a less ionic (or more metallic) bond, and therefore, others [34,35,36] have hypothesized that selenium SAMs offer better conduction properties. l-selenomethionine and Se-methylseleno-l-cysteine can be adsorbed on S-Au surfaces by replacing sulfur with selenium in a conjugated molecule. In the present study, we report a novel method of determining l-selenomethionine and Se-methylseleno-l-cysteine: using a SAM-covered gold electrode that chemically reacts as a selective sensor with thiol groups and is coupled with HPLC. Electrochemical flow cell devices were designed to study l-selenomethionine and Se-methylseleno-l-cysteine flow through gold and modified gold electrode electrochemical processes. The optimal experimental conditions for determining l-selenomethionine and Se-methylseleno-l-cysteine in pharmaceutical products are described in this paper.

## 2. Experimental Section

### 2.1. Apparatus and Materials

All electrochemical measurements were performed with a potentiostat-galvanostat (SP-150; Bio-Logic SAS, Claix, France) with a conventional three-electrode configuration with gold and thiol-compound-modified gold as a working electrode. Potentials were measured versus an Ag/silver chloride (AgCl) electrode (RE-1; Bioanalytical Systems, West Lafayette, IN, USA), and a platinum wire was used as the auxiliary electrode. An HPLC system (LC-10 ADvp; Shimadzu, Kyoto, Japan) with an injection valve (Rheodine 7125; Sigma-Aldrich, St. Louis, MO, USA) with a 20-mL sample loop was coupled to an amperometric detector (Decade SDC; Antec Leyden B.V., Zoeterwoude, The Netherlands). The flow cell was designed with the following electrodes: an Ag/AgCl/0.1 M potassium chloride (KCl) reference electrode (Bioanalytical Systems), a platinum auxiliary electrode and a gold or a thiol-compound-modified gold electrode (length: 8 cm; internal diameter (i.d.): 3 mm) as the working electrode for detecting l-selenomethionine and Se-methylseleno-l-cysteine. The thiol compounds were l-selenomethionine, Se-methylseleno-l-cysteine and 2-mercapto-5-thiazoline (TCI; Tokyo, Japan); 2,4-thiazolidinedione (Acros Organics; Geel, Belgium); 2-mercaptothiazoline (Alfa Aesar; Ward Hill, MA, USA); l-cysteine (Sigma-Aldrich); and thioglycolic acid (E. Merck Chemical Co.; Darmstadt, Germany). The commercial samples containing l-selenomethionine and Se-methylseleno-l-cysteine supplements were produced abroad, but purchased locally: Vitacost Selenium Select^®^ (selenium 200 mcg), Solgar yeast-free selenium (selenium 100 mcg), Irwin Naturals Living Green Liquid-Gel Multi (selenium 75 mcg), LifeExtention (selenium 200 mcg) and Jarrow Formulas (selenium 200 mcg). All other reagents were of analytical grade.

### 2.2. Determining l-Selenomethionine Using Cyclic Voltammetry

Cyclic voltammetry (CV) was done in phosphate buffer (pH 2.23–6.57), acetate buffer (pH 4.18), Britton and Robison buffer (pH 2.46–10.45) and lithium perchlorate (LiClO_4_) solutions as supporting electrolytes for a gold electrode. CV potentials ranged from −0.4 V to +1.5 Vat a scan rate of 25 mV·s^−1^. The CV data were calculated using SP-150, Bio-Logic SAS and EC-Lab^®^ software. The standard stock solution was prepared by dissolving the appropriate amount of l-selenomethionine in methanol.

### 2.3. Preparing Thiol-Modified-Au Electrodes

The gold wire electrode was cleaned then placed in the tube containing thiol compound solution, deoxygenated by purging with nitrogen for 5 min. The different thiol-modified-Au electrodes were electrolytically plated with a thiol compound (2-mercapto-5-thiazoline, 2-mercaptothiazoline, l-cysteine, thioglycolic acid or 2,4-thiazolidinedione) from 10 mL of 0.1 M acetate buffer (pH 4.23), 0.01 to 1.6 mM thiol solution, respectively, and the potential was cycled between 0.0 V and 1.5 V (versus an Ag/AgCl reference electrode) with a scan rate of 25 mV·s^−1^ until the CVs showed no further change.

### 2.4. Construction of a Voltammetric Sensor for Liquid Chromatography

The flow cell was designed with the following electrodes: an Ag/AgCl/0.1 M KCl reference electrode, a stainless steel auxiliary electrode and a modified thiol/Au (length: 8 cm; i.d.: 0.3 mm) as the working electrode for detecting l-selenomethionine and Se-methylseleno-l-cysteine. The amperometric detection was achieved in a homemade flow-through cell prepared in our laboratory as previously described [37]. A flow-through electrolysis cell was used for DC-mode amperometric detection. Reversed-phase HPLC was done on a column (250 mm × 4.6 mm) (LiChroCart^®^; Merck, Darmstadt, Germany) eluted with methanol and water (10:90 v/v) containing 1.0 mM of KH_2_PO_4_ (pH 3.89) as the mobile phase, at a flow rate of 1.0 mL·min^−1^. It was examined using an ultraviolet (UV) detector set at 248 nm. The electrochemical detector was operated between +0.6 V and 1.3 V for gold and thiol-modified-Au electrodes.

### 2.5. Application to Commercial Selenium Supplement Drugs

A set of standard solutions was produced by diluting aliquots of the stock solutions with methanol to 10 mL in calibrated flasks. Taking into account the l-selenomethionine or Se-methylseleno-l-cysteine content of the selenium supplement drug samples (approximately 0.008–0.02 g), the latter were accurately weighed in a 55-mL polyfluoroalkoxy bottle diluted to about 10 mL of methanol:water (1:1, v/v), put into a microwave (MARS 6 System; CEM Corp., Matthews, NC, USA), dissolved and then centrifuged. The supernatant was transferred into a 10-mL calibrated flask. An aliquot of the solution was filtered through a 0.45-µm membrane filter before it was analyzed using HPLC. A chromatograph was obtained using 20 mL of the prepared standard solution under the operating conditions described above.

## 3. Results and Discussion

### 3.1. Voltammetric Behavior of l-Selenomethionine on Au Electrodes

**Figure 1:**
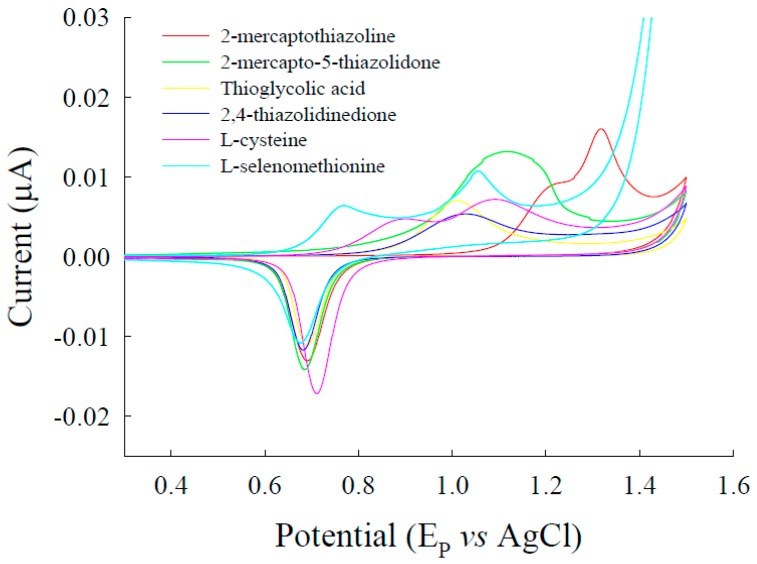
Cyclic voltammograms of l-selenomethionine on bare gold electrodes and on gold electrodes modified by different thiol compounds (0.074 mM) (thiol/Au) at 25 mV/s in acetate buffer (pH = 4.23).

The anodic peak potential and current of l-selenomethionine in 0.1 M acetate buffer (pH 4.23) of the bare gold electrode were (0.823 V, 6.67 µA) and (1.11 V, 9.49 µA), respectively (Figure 1). The anodic peak potentials and currents of l-selenomethionine were for 2-mercaptothiazoline modified gold (1.21 V, 8.87 µA) (1.342 V, 16.0 µA), 2-mercapto-5-thiazoline modified gold (1.12 V, 12.0 µA), l-cysteine-modified gold (0.891 V, 4.71 µA) (1.09 V, 7.19 µA), thioglycolic acid-modified gold (1.00 V, 7.03 µA) and 2,4-thiazolidinedione-modified gold electrodes (1.02 V, 5.36 µA) (Figure 1).

**Figure 2:**
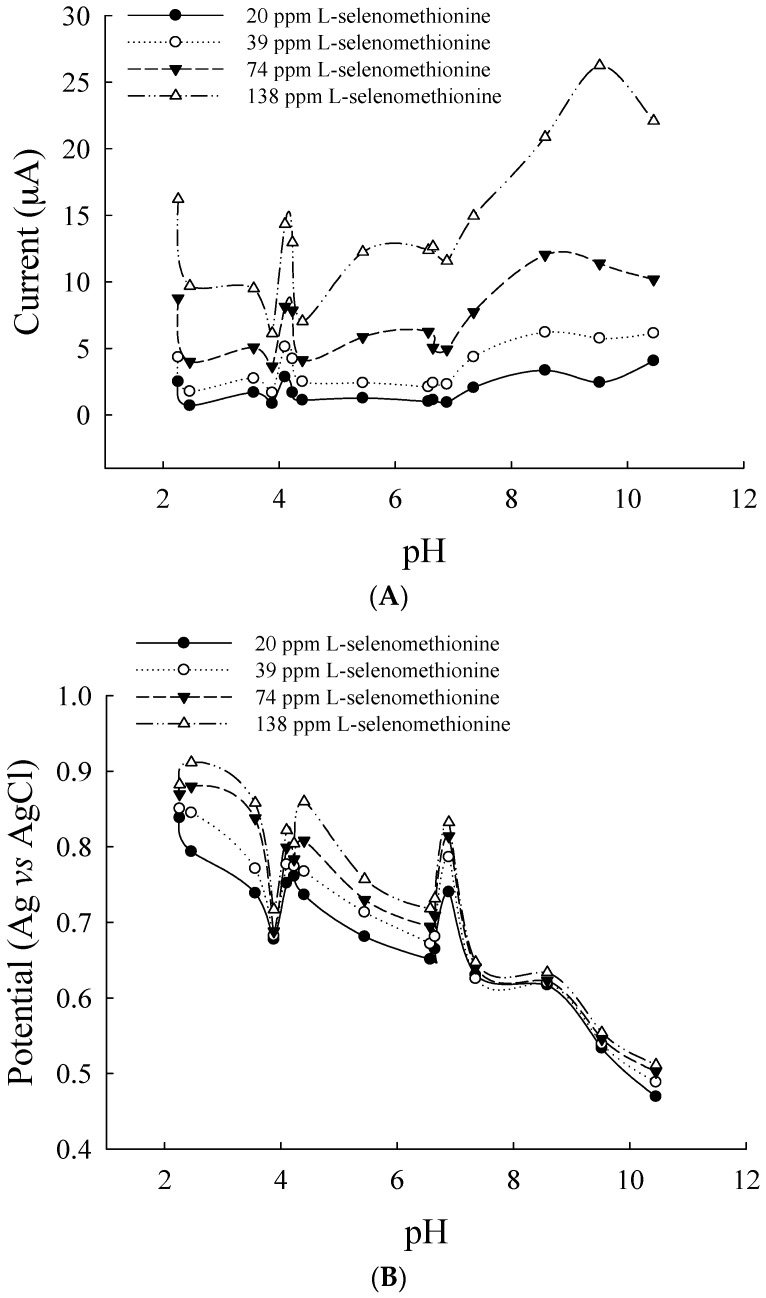
Effect of pH values on anodic peak in Britton and Robison buffer (pH 2.46–10.45) containing 20–138 mg·L^−1^ l-selenomethionine. (**A**) The relationships between peak currents and pH and (**B**) the relationships between potential and pH.

Acid-based equilibrium directly affected the electroactive species; changes in peak current, potential and pH can also occur because of chemical reactions [22,23]. l-selenomethionine was protonized in the pH < 2.1 region because of acid-catalysis (Figure 2A). In the pH 2.2–4.0 region, the first current peak was almost constant, but when the pH was >3.2, the current increased with increasing pH values: the highest peak was at pH 4.0. In the pH 5–7 region, the unprotonized form of l-selenomethionine underwent uncatalyzed changes that were similar to the isoelectric points of cysteine and l-methionine at pH 5.05 and pH 5.74, respectively. The peak potential decreased with increasing pH values, and the shift was less positive (Figure 2B). The two approximately linear portions (plotted for (pK −1) > pH > (pK +1)) intersect at a pH value corresponding to pK. Based on l-selenomethionine pKa values in acid (pK 2.15) and alkaline (pK 8.94), different species (protonized and unprotonized) might be present in aqueous solutions. The peak currents are higher at pH 2.5 and pH 4.2, and at pH 4.2, the peak potential was the lowest within the acidic range. Because acidic solution is suitable for HPLC column analysis, all of the following experiments were done at pH 4.2. However, in inorganic redox systems, there are no proton transfers (only electron state transitions); thus, the electron transition was not significantly affected by changes in pH.

### 3.2. Optimal Conditions for a Flow-Through Voltammetric Detector

**Figure 3:**
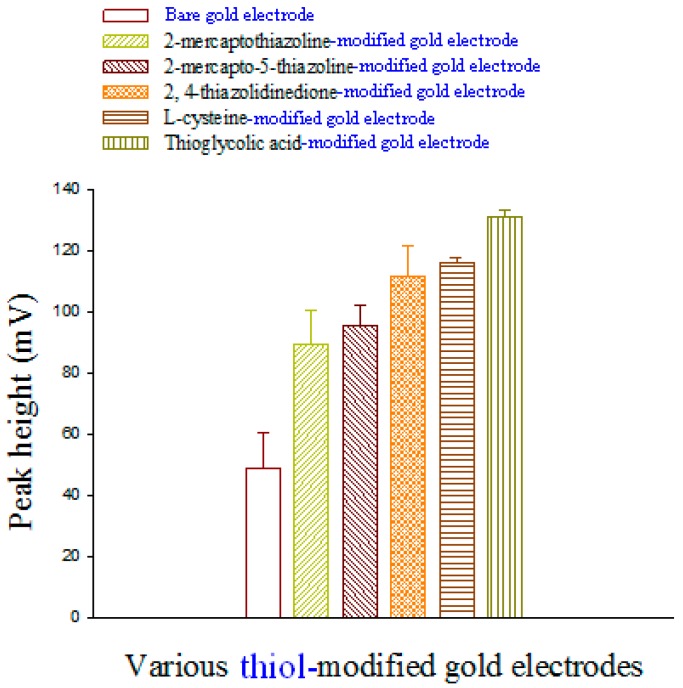
A comparison of an l-selenomethionine (0.4 mg·L^−1^) bare gold electrode and various l-selenomethionine (0.4 mg·L^−1^) thiol/Au electrodes in a flow-through voltammetric sensor; the mobile phase is methanol-water (10:90, v/v).

**Figure 4:**
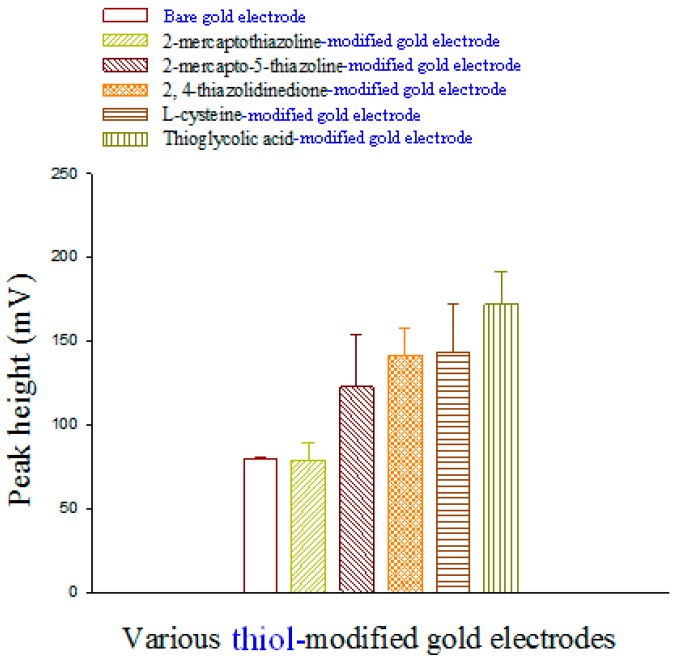
A comparison of a bare Se-methylselenocysteine (0.4 mg·L^−1^) gold electrode and various Se-methylselenocysteine (0.4 mg·L^−1^) thiol/Au electrodes in a flow-through voltammetric sensor; the mobile phase is methanol-water (10:90, v/v).

Methanol-water that contained various ratios of 0.1 mM phosphate buffer (pH 3.5–4.2) was prepared. Experiments showed that l-selenomethionine was retained for 3.62 min and that Se-methylseleno-l-cysteine was retained for 2.77 min, after which, baseline separation was recovered. Methanol-water (10:90, v/v) that contained phosphate buffer (pH 3.92) was found to be the best eluent for sensitivity higher than that of other eluents. Therefore, phosphate-buffered solution was chosen for determining l-selenomethionine and Se-methylseleno-l-cysteine. To compare the electroanalytical utility of thiol/Au electrodes, we used liquid chromatography with electrochemical detection (LC-ECD) to measure the l-selenomethionine and Se-methylseleno-l-cysteine on thiol/Au electrodes and bare gold electrodes in methanol-water (10:90, v/v). The peaks of l-selenomethionine and Se-methylseleno-l-cysteine on thiol/Au electrodes are higher than that of the bare gold electrode (Figure 3 and Figure 4). The sensitivity of thioglycolic acid (TGA) is greater than that of the other thiol/Au electrodes. Because the TGA/Au electrode has only one peak, the interference is less than that of the other modified electrodes, and the catalytic effect (the current) is greater (Figure 1). Therefore, we used the TGA/Au electrode to determine l-selenomethionine and Se-methylseleno-l-cysteine in commercial selenium supplements.

**Figure 5:**
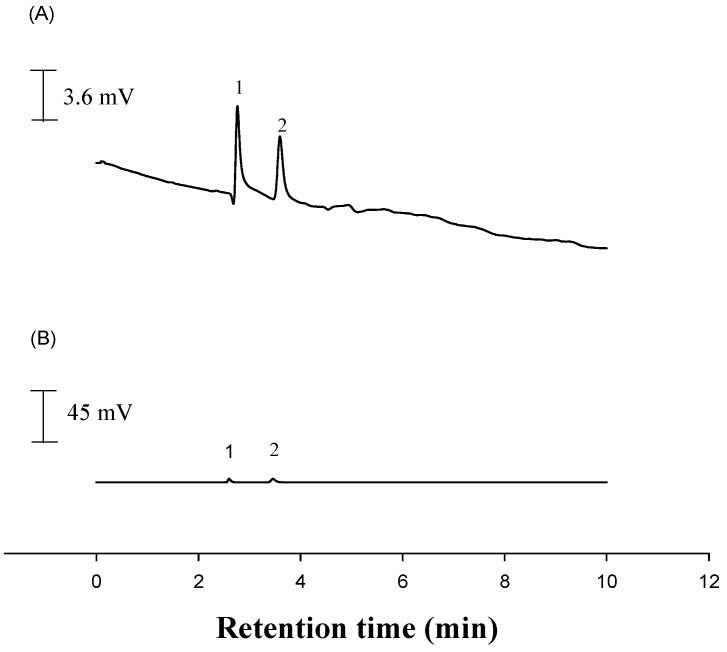
Chromatograms obtained using (**A**) liquid chromatography with electrochemical detection (LC-ECD) (0.4 mg·L^−1^) and (**B**) LC- ultraviolet detection (UVD) (20 mg·L^−1^). Peak 1 is Se-(methyl)seleno-l-cysteine; Peak 2 is l-selenomethionine, both in standard solution; LC-ECD at the TGA/Au electrode (1.2 V); LC-UVD at 248 nm. Analysis conditions are identical to those listed in Figure 3.

The voltammetric detector was operated at +1.2 V. Using the injection valve, 20 μL of the prepared standard solutions was chromatographed under the operating conditions described above. The lower limit of quantitative detection in our method was approximately 0.16 ng for l-selenomethionine and 0.07 ng for Se-methylseleno-l-cysteine. The calibration graph plots obtained by plotting the peak area against the concentrations of l-selenomethionine and Se-methylseleno-l-cysteine show good linearity over the range 10–1600 ng·mL^−1^ and 50–1600 ng·mL^−1^, respectively. The regression equations were *y* = 157*x* + 4.5 (r = 0.9987) for l-selenomethionine and *y* = 322*x* − 7.8 (r = 0.9996) for Se-methylseleno-l-cysteine. Chromatograms were obtained using LC-ECD and LC- ultraviolet detection (UVD) (wavelengths at 248 nm) (Figure 5A,B). LC-ECD is more specific and sensitive than LC-UVD.

### 3.3. Accuracy and Precision

**Figure 6:**
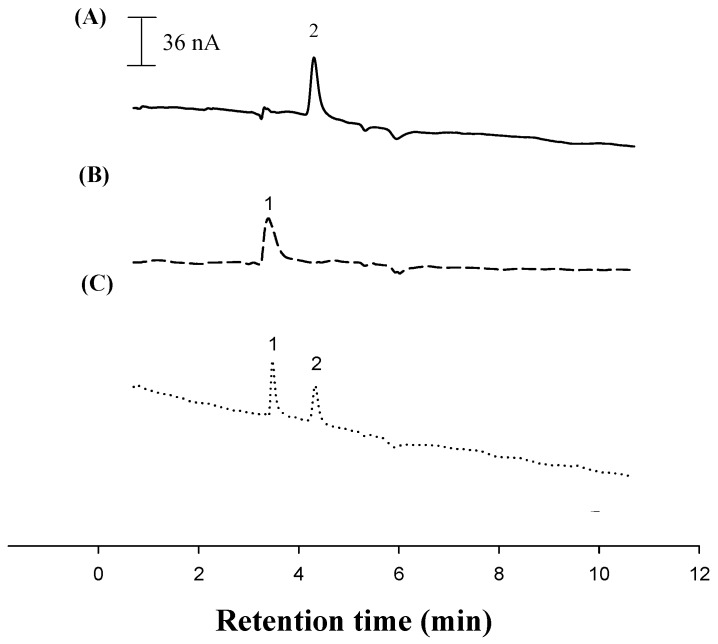
Chromatograms: Peak 1 is Se-(methyl)seleno-l-cysteine, and Peak 2 is l-selenomethionine obtained using a TGA/Au electrode. (**A**) The solid line is commercial l-selenomethionine-containing supplements; (**B**) the broken line is commercial Se-(methyl)seleno-l-cysteine-containing supplements; and (**C**) the dotted line is the standard solutions. Analysis conditions are identical to those listed in (Figure 3).

To test the applicability of the developed TGA/Au electrode, a commercial selenium supplement was analyzed using the standard addition method. Recovery tests were done on commercial selenium supplements to evaluate the reproducibility and accuracy of the proposed LC-ECD and LC-UVD methods. Each sample was diluted with methanol and water, and they were spiked with different concentrations of l-selenomethionine and Se-methylseleno-l-cysteine. The recoveries ranged from 97%–104% (LC-ECD) (Table 1). Hence, in this study, determining the concentration of selenoamino acids in commercial selenium supplements was done using a standard additions procedure (Table 2). These results agreed with those obtained using an LC-UVD. The sensitivity of LC-ECD was 680–1280-times greater than that of LC-UVD. In Samples 4 and 5, no Se-methylseleno-l-cysteine was detected because it was below the LC-UVD level of detection. Representative LC-ECD chromatograms (Figure 6A–C) for the selenium supplement sample are comparable to a chromatogram of pure standard. Inorganic selenium has various oxidation states (VI, IV, 0, −II) over a wide range of pH, and it appears primarily as selenite (Se (IV)) and selenate Se (VI)). The electrochemical behavior of inorganic selenium species, e.g., Se (IV) and Se (VI), requires Se (VI) to be reduced to Se (IV) and then done using stripping voltammetric techniques [38]. Sodium selenite is a major component of some commercial supplements, e.g., Twinlab capsules (selenium 250 mcg) and Allergy Research Group selenium solution (selenium 100 mcg), but cannot be determined in our LC-ECD conditions. The diselenide forms cannot be detected in commercial products either, because they are first cleaved to yield an Se-Au adsorbate, and then, bulk selenocysteine is reduced on the modified electrode [39].

## 4. Conclusions

We have developed a sensitive, green electrochemical procedure for determining organic selenium using a flow-through voltammetric sensor. It exhibits a good analytical performance using a thiol/Au electrode for the LC-ECD of l-selenomethionine and Se-methylseleno-l-cysteine with a low limit of quantitation, a rapid response, a satisfactory linear range and good stability and selectively. The presented electrode was simultaneously used to determine l-selenomethionine and Se-methylseleno-l-cysteine in a commercial selenium supplement.

## Figures and Tables

**Table 1:** Recovery of l-selenomethionine and Se-methylseleno-l-cysteine in spiked commercial selenium supplements using liquid chromatography with electrochemical detection (LC-ECD).

Samples	Added (mg·L^−1^)	Found ^a^(mg·L^−1^) Mean ± SD ^b^	Recovered (%)
l-selenomethionine			
Sample 1	0.5	0.486 ± 0.007	97.3
	1.0	1.014 ± 0.018	101
	2.0	1.985 ± 0.024	99.2
Sample 2	0.5	0.488 ± 0.046	97.6
	1.0	1.045 ± 0.069	104
	2.0	2.002 ± 0.040	100
Sample 3	0.5	0.507 ± 0.068	101
	1.0	1.036 ± 0.064	103
	2.0	1.993 ± 0.022	99.6
Se-methylseleno-l-cysteine ^b^			
Sample 4	0.5	0.499 ± 0.023	99.8
	1.0	1.011 ± 0.031	101
	2.0	1.995 ± 0.013	99.7
Sample 5	0.5	0.549 ± 0.023	105
	1.0	1.031 ± 0.014	103
	2.0	2.034 ± 0.032	103

Data are the means of three measurements. ^a^ Number of determinations (N = 3), ^b^ SD, standard deviation.

**Table 2:** Analytical results of determination of l-selenomethionine and Se-methylseleno-l-cysteine in commercial selenium supplement products using liquid chromatography with electrochemical detection (LC-ECD) and ultraviolet detection (LC-UVD).

Samples	l-selenomethionine		Se-methylseleno-l-cysteine ^b^	
	Concentration (%, w/w) N = 3 ^a^		Concentration (%, w/w) N = 3 ^a^	
	LC-ECD	LC-UV	LC-ECD	LC-UV
Sample 1	0.035 ± (3.4)	0.036 ± (2.8)	- ^c^	- ^c^
Sample 2	0.101 ± (1.4)	0.102 ± (2.0)	- ^c^	- ^c^
Sample 3	0.131 ± (3.0)	0.133 ± (3.1)	- ^c^	- ^c^
Sample 4	- ^c^	- ^c^	0.138 ± (2.5)	- ^c^
Sample 5	- ^c^	- ^c^	0.875 ± (0.4)	- ^c^

^a^ Number of determinations (N = 3). ^b^ Values in parentheses indicate relative standard deviation. ^c^ Not determined.
